# Botulinum Toxin and Percutaneous Needle Electrolysis for the Treatment of Chronic Masticatory Myalgia

**DOI:** 10.3390/toxins15040278

**Published:** 2023-04-10

**Authors:** Luis-Miguel Gonzalez-Perez, Ramon Vera-Martin, Enrique Montes-Latorre, Eusebio Torres-Carranza, Pedro Infante-Cossio

**Affiliations:** 1 Department of Oral and Maxillofacial Surgery, Virgen del Rocio University Hospital, 41013 Seville, Spain; lumigon@telefonica.net (L.-M.G.-P.); veramartinramon@gmail.com (R.V.-M.); drtorres@clinicatorrescarranza.es (E.T.-C.); 2 Department of Surgery, School of Medicine, University of Seville, 41009 Seville, Spain; 3 Department of Clinical Neurophysiology, Virgen del Rocio University Hospital, 41013 Seville, Spain; enriquemonteslatorre@hotmail.com

**Keywords:** long-term masticatory myalgia, temporomandibular disorders, pain management, electromyography, botulinum toxin type A, percutaneous needle electrolysis, randomized controlled trial

## Abstract

Botulinum toxin type A (BTA) is applied in muscle hyperactivity disorders and injected into affected muscles, producing deep and persistent muscle relaxation. Several multidisciplinary groups investigated the treatment of temporomandibular disorders for several years, and there is currently some data on the beneficial effects of BTA in specific cases of chronic masticatory myalgia. Percutaneous needle electrolysis (PNE), which applies a low-intensity galvanic current to promote tissue regeneration, has been shown to be effective in reducing pain and improving masticatory function. The purpose of this study was to investigate the efficacy and safety of BTA and to assess whether its application in patients with localized masticatory myalgia can significantly reduce pain and improve function compared to a group treated with PNE. Fifty-two patients with long-term refractory masticatory myalgia were randomly assigned to two groups. The BTA group (*n* = 26) received a bilateral botulinum toxin injection and the PNE group (*n* = 26) received percutaneous electrolysis. The dose of BTA injected was 100 units distributed among the main primary masticatory muscles, and PNE was administered at 0.5 mA/3 s/3 consecutive times in a single session. Patient assessments were performed prior to treatment and one, two, and three months after treatment. The results revealed good therapeutic response in both groups. In the long term, both BTA and PNE showed high efficacy and safety in reducing pain and improving muscle function for the treatment of chronic masticatory myalgia. This improvement was sustained over a three-month period in both groups. Therefore, the use of BTA and PNE could be considered a valid and safe therapeutic alternative among the available options to treat refractory and localized masticatory myalgia when a better therapeutic response is expected as it demonstrated high efficacy.

## 1. Introduction

Masticatory myalgia (MM) is a common musculoskeletal condition produced by a sustained and persistent contraction of a group of muscles responsible for mandibular movement, whose main characteristic feature is the frequent occurrence of chronic pain in multiple trigger points (TrPs). These TrPs correspond to hyperirritable spots in skeletal muscle associated with palpable nodules in taut bands of muscle fibers within the affected muscles, accompanied by regional muscle pain, or referred pain, to the nearby masticatory muscles or the temporomandibular joint (TMJ). On examination, TrPs may be identified within a tight band of the involved muscle, which may cause referred pain and a local twitch reaction upon palpation. Regional discomfort may be associated with muscle stiffness, limitation of movement, weakness, and, in some cases, autonomic dysfunction [1,2,3,4]. Consequently, these disabling symptoms can significantly affect many domains of quality of life. The pathophysiological basis of this temporomandibular disorder depends on the activation of TrPs, which can be elicited by multiple factors including, among others, acute or chronic muscle overload, psychological distress, homeostatic imbalances, and direct trauma to the area. Some of these processes complement each other, and long-term MM is probably the consequence of the sum of more than one of these mechanisms [5].

Management of MM consists of alleviating the patient’s pain and achieving a correct relationship between the TMJ and the affected muscles, with an adequate range of motion. Several treatments have been proposed for MM. The main management modalities are patient education and control of predisposing factors; analgesics, pharmacological therapies, such as myorelaxants and antidepressants; occlusion splints; various physiotherapy treatments; dry needling; ultrasound; transcutaneous electrical nerve stimulation; psychotherapy; or TrP injections and wet needling of different substances [2,5,6,7]. Although in a large number of patients a satisfactory reduction in pain can be achieved after one or more conservative treatment methods, there remains a significant group of refractory cases that should be directed towards minimally invasive procedures, with dry needling being one such technique used to inactivate TrPs without the injection of any substance [8]. Several studies have shown that dry needling provides effective pain relief and short-term muscle functional recovery, and have reported its safety, efficacy, and low cost [9,10]. Dry needling can be combined with an electrical current to provide other management modalities. Percutaneous needle electrolysis (PNE) is a minimally invasive treatment consisting of muscle puncture to apply galvanic microcurrents through a specific medical device with a puncture needle like that used in dry needling with the aim of activating regenerative processes in injured muscles. PNE has been shown to be effective in reducing pain and improving masticatory function [11,12,13], possibly because this technique combines mechanical stimulation (needle) and electrical stimulation (galvanic current) [11]. Furthermore, PNE treatment has been reported to be more cost-effective than dry needling [14].

For decades, botulinum toxin A (BTA) has been applied in a multitude of disorders related to muscle hyperactivity and injected into the affected muscles, producing chemical denervation and deep and persistent relaxation [15]. In recent years, several multidisciplinary groups have investigated the treatment of temporomandibular disorders, and there is currently some data on the beneficial effects of BTA in specific cases of refractory pain in the masticatory muscles [2,6,16,17,18], although more studies should be carried out to recommend its appropriate use in order to avoid undesirable therapeutic effects. At the same time, given the wide variety of treatment modalities described for the management of chronic MM, it is necessary to know the actual role of BTA injection compared to other minimally invasive techniques. The purpose of this study was to investigate the efficacy and safety of BTA and to evaluate whether its application in patients with chronic localized MM can significantly reduce pain and improve function compared to patients treated with PNE. 

## 2. Results

**Figure 1:**
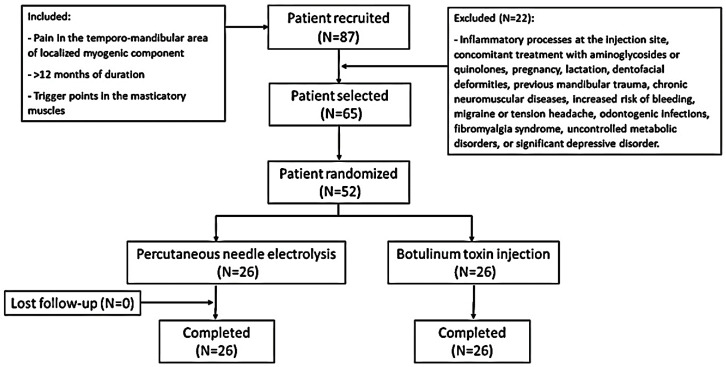
Flow chart of the study.

Initially, 87 patients were recruited after ruling out other medical pathologies by orthopantomography and magnetic resonance imaging (MRI). After an initial medical consultation, a total of 65 patients with temporomandibular pain with a localized myogenic component of a duration of more than 12 months, without signs of internal joint damage and with a TrP in the masticatory muscles, were selected (Figure 1). Finally, 52 patients were included and randomly assigned to each study group (*n* = 26 in each group). All of the patients in both groups completed the trial. Table 1 shows the demographic characteristics and description of variables of all the participants at baseline. The two groups had similar mean age distributions (BTA group = 39.11 ± 9 years, range = 22–54 years; PNE group = 41.96 ± 9.88 years, range 25–62 years). There was a female predominance in the female/male ratio (BTA group = 2.7/1; PNE group = 2.2/1). At the start of the study, no statistically significant differences were found for any of the variables. The radiological and MRI findings revealed no significant changes. Electromyography (EMG) detected no differences between masticatory pain patterns in the two groups prior to therapeutic administration.

Table 2 shows the intergroup and intragroup analysis statistics for each group on the different study days, presented as mean (M) ± standard deviation (SD). In the intergroup analysis of the three measurements up to day 90, no statistically significant differences were found between the BTA and PNE groups. When evaluating the intragroup differences at each control point, the changes in values were suggestive and statistically significant for the BTA and PNE groups with respect to day 0 in all of the variables studied (pain intensity, maximum interincisal opening (MIO), lateral movements, i.e., right and left, and protrusive movements). 

Table 3 shows the results of the comparative analysis between two consecutive control checkpoints. Statistically significant differences in the reduction in pain intensity and MIO were found in the measurements between days 0 and 28 in both of the study groups. The values remained stable until day 90. 

The values obtained on the 100-point questionnaire improved statistically significantly between day 0 and day 90 in both groups (Table 4). The assessment of the efficacy and tolerability outcomes for both the patient and the observer did not reflect significant differences at any checkpoint.

Four patients reported mild side effects, all in the PNE group, consisting of pain (two cases) and bruising at the puncture site (two cases). None of the observed adverse effects prevented the continuation of the study.

## 3. Discussion

In this randomized clinical trial, we aimed to evaluate the use of BTA and PNE in a group of patients with chronic MM, who were similar in terms of mean age, gender distribution, pain patterns, range of mandibular movements, and electromyographic recordings. When comparing the two therapeutic tools with each other, neither showed significant superiority over the other in any of the variables nor in the efficacy and tolerance of the patient and the observer. However, four cases of mild side effects were detected in the PNE group, which did not prevent the study from being carried out. In general, our findings revealed that statistically significant positive therapeutic responses were obtained in each group. Compared to pre-treatment baseline values, both BTA and PNE were effective procedures that significantly improved pain and masticatory muscle function. The results were significant in the first month, although improvement in the different variables was maintained during the three months of the study in each group. 

Chronic MM is a debilitating health condition and one of the leading causes of consultation in craniofacial pain [19]. It causes limitations in activities of daily living and has serious social and occupational consequences, affecting the patient’s quality of life and limiting work performance [15]. Despite the high prevalence of temporomandibular disorders, there is no general consensus on their treatment as they may respond poorly to conservative therapeutic modalities based on occlusion splints, physiotherapy measures, analgesics, non-steroidal anti-inflammatory drugs, or muscle relaxants [1,2]. In cases refractory to these conservative therapies, other treatments are considered, such as those that we aimed to evaluate in this trial. Our results suggest that the use of BTA and PNE can be considered valid therapeutic options to treat refractory and localized MM and can be recommended as safe therapeutic alternatives. In addition, improvements in masticatory muscle function and in the 100-point test score were similar in the BTA and PNE groups, resulting in an increase in the perceived quality of life of patients.

In 2014, some diagnostic categories in the RDC/TMD (Research Diagnostic Criteria for Temporomandibular Disorders) were eliminated, while others such as localized myalgia were included. In the new DC/TMD classification (Diagnostic Criteria for Temporo-Mandibular Disorders for Clinical and Research Applications) [20], which is one of the most widely used classifications in clinical practice, pain is an important component of diagnosis that acquires value as a central symptom of these disorders. New specific terms, such as local MM, have replaced what was previously known as the more general term “myofascial pain syndrome”. Currently, MM has two possible diagnostic categories depending on whether the pain is localized or referred to other areas [21], which was taken into account during the selection of patients in our study. In view of our results, we believe that localized or referred myalgia, characterized by localized myogenic pain on palpation that extends within the boundaries of the affected masticatory muscles, should be the main indication for the use of these procedures. In our case, pain did not extend to areas outside the boundaries of the affected muscle, and the physical presence of a hypersensitive, indurated nodule within a muscle mass of normal consistency was the most frequent finding associated with a TrP on physical examination, and its palpation provoked pain directly in the affected area. The treatment of chronic MM in adults is challenging due to its technical difficulties and the high incidence of cases, so we believe that these procedures should be performed after a correct diagnosis has been made. In our study, the term myalgia describes pain of muscular origin that worsens with functional or parafunctional movements. Physical examination should confirm the location of pain in the masseter, temporalis, or pterygoid muscles. The different types of myalgia, determined by DC/TMD classification, differ fundamentally in their extent, although they retain the general characteristics described [18].

We designed a randomized controlled study comparing two minimally invasive emergent interventions. As in previous studies, an adequate sample size of 26 subjects per group was calculated [2,8,10]. We established this estimate by taking into account the required change in VAS pain scores and the existing standard deviation of the results. All of the participants had the same pain patterns and range of mandibular movements so that comparisons could be made between the groups in terms of the results obtained. The demographic characteristics and description of variables at baseline were similar. Furthermore, data collection was performed at three standardized checkpoints during the postoperative period, which facilitated comparisons with the preoperative baseline and between the BTA group and the PNE group. PNE is a virtually painless and minimally invasive technique with favorable therapeutic results in previous studies [11], based on the use of galvanic microcurrents through a dry needle guided by EMG monitoring for greater precision in order to identify the muscle masses to be treated and to detect specific patterns of normality or muscle involvement, both at rest and during voluntary activity. PNE was performed by applying a low-intensity galvanic discharge to the TrPs, at 0.5 mA/3 s/3 consecutive times in a single session, with the aim of inactivating them and accelerating regeneration of the damaged muscle.

Although BTA is an effective treatment for masticatory muscle pain, few studies uniformly support it in the orofacial area, undoubtedly due to the heterogeneity of the samples studied to date [22,23,24,25,26,27]. Freund and Schwartz [28] were the first to suggest the benefits of BTA for the treatment of myogenic pain, reporting that 90% of cases with cervicofacial pain showed improvement in pain and function after injection. Since then, several case series and cohort studies have suggested different techniques, doses, and dilutions of BTA and have, in general, also shown a long-term therapeutic response in temporomandibular disorders but with some conflicting results [4,29]. In line with previous studies, our results show that BTA could be a therapeutic indication for clinical cases that do not obtain significant symptomatic relief with standard conservative treatments [18]. 

The efficacy of myofascial pain relief following BTA injection has not yet been established in the literature. We believe that one of the main reasons for this controversy is related to the wide disparity in the inclusion and exclusion criteria used in the reviewed studies [2,6,14,30,31,32,33]. Because there are several diagnostic criteria for chronic masticatory pain, some studies have applied inclusion criteria that could have been carried out for the exclusion of patients in other studies. This could result in an underestimation of the true effect size by including patients whose condition is doubtful to improve following treatment and may give the ambiguous appearance of a failed trial when, in reality, a subgroup of the study may have experienced real benefits. In a previous randomized clinical trial conducted by our group, we reported a clear improvement in refractory MM, with a significant reduction in masticatory muscle pain when injecting BTA in cases of localized myalgia [2]. This positive response, although significant, has not been observed with the same intensity in patients with referred myofascial pain irradiating to regions away from the masticatory muscles. Therefore, it is useful to use reliable diagnostic criteria for temporomandibular disorders and the classification of refractory MM into subgroups corresponding to the type of pain location when using BTA in those patients in whom a better response to treatment is expected. In our case, the recording of EMG activity prior to performing the injection is useful to recognizing the muscles to be treated. This is one of the purposes of this study, as chronic MM has a very good therapeutic response to BTA in small doses if applied correctly in selected appropriate cases.

In addition, it is important to highlight that the EMG recording we performed before treatment and the use of specific needle electrodes for BTA injections are useful and necessary procedures to identify the muscle masses to be treated and to avoid administration errors and side effects. In our literature review, we also found significant variation in the doses of BTA used and the muscles injected [18]. In our study, a dose of 100 units of BTA was used and distributed bilaterally in the masseter, temporalis, and lateral pterygoid muscles. Similarly, a single injection of BTA has been reported to have long-term beneficial therapeutic effects [34]. The onset of pain reduction after BTA administration is expected relatively late, as in previous studies the results were significant at 7 days [2]. These changes are especially evident in patients with a chronic and localized pain pattern, probably due to the gradually accumulative analgesic property of the toxin, which reaches a peak of efficacy after one week of continuous muscle relaxation. This analgesic effect of BTA appears after a period of constant and sustained relaxation of painful muscle fibers, as is the case with the most common conservative treatment modalities. The effect of BTA on MM is maintained for at least 4–6 months in appropriately selected cases, thus showing that analgesic action persisted during the 90-day follow-up of our study [2,7,16].

Finally, our patients were evaluated taking into account the contraindications of treatments. The efficacy and tolerability were the same in both groups as estimated by the patient and the observer. As with any minimally invasive technique, both BTA and PNE were well tolerated, with no significant contraindications. No side effects were detected with BTA, while four cases of self-limited pain and bruising at the puncture site were reported in the PNE group. In our experience, it is useful and convenient to localize the affected muscles by EMG, so in this study we used needle electrodes that allow BTA injection at the same time. Multipoint injection achieves more uniform contact between BTA and the innervated muscle areas, which is especially advantageous when infiltrating larger muscles. It is important to bear in mind that local diffusion of BTA may depend on the volume and dose administered and that large volumes may impair the integrity of the muscle fascia, so this is another aspect we have taken into account to minimize side effects in our study. It is generally accepted that low doses of botulinum toxin should be administered to limit the associated adverse effects [7]. 

Our study had some limitations, such as the inconvenience of blinding the study subjects, which limits the validity of the measurement of the results obtained. Although three months of follow-up is an adequate time to evaluate the results, studies that consider longer evaluation periods should be conducted. The effect of psychosocial variables on temporomandibular disorders should also be considered, as they may act as factors in the chronification of pain [1,2,5,8,35]. Although several studies have demonstrated the benefits of BTA use, there is no general consensus on the therapeutic advantage of this tool in the management of temporomandibular disorders, and more randomized clinical trials with larger samples, minimal bias, and longer follow-up periods should be conducted. The best site to inject the toxin and the optimal dose must be determined, and BTA cost-effectiveness studies must be conducted to estimate whether the cost–benefit ratio is clinically acceptable. Epidemiological studies on the prevalence of patients with localized MM are scarce, and more valid data are needed in the context of a public health system such as ours [2,8,10]. Therefore, its cost should be considered in comparison with other less costly conservative measures, such as PNE [14]. However, considering costs alone may not be sufficient to make definitive therapeutic decisions on whether it is a specific and justifiable treatment because other factors also contribute substantially, such as the social and occupational burden associated with chronic MM.

## 4. Conclusions

The main objective of this 3-month prospective clinical trial was to evaluate the efficacy and safety of BTA in the treatment of refractory and localized MM compared to another minimally invasive treatment modality, such as PNE. In our study, these procedures were performed with the help of EMG prior to therapeutic administration. The results obtained showed a significant reduction in pain and an improvement in masticatory function. Our study revealed that it is possible to achieve satisfactory mouth opening and free and painless mandibular movement using both BTA and PNE in the treatment of chronic masticatory pain, with little or no adverse effects. The optimal use of BTA and PNE in patients with localized myalgias in the masticatory muscles could contribute to improving the efficiency of care in healthcare systems. Therefore, the use of BTA and PNE could be considered a valid and safe therapeutic alternative among the available options to treat refractory and localized MM when a better therapeutic response is expected, as both demonstrate high efficacy.

## 5. Materials and Methods 

### 5.1. Standard Protocol Approvals and Patient Consents

This study was approved by the Research Ethics Committee of Virgen del Rocio University Hospital (Spain) (IRB number: PI 16/970), and the Declaration of Helsinki guidelines were followed. All of the participants were informed of the nature of the study and their written consent was obtained.

### 5.2. Study Design and Subjects

We conducted a single-center, randomized clinical trial between June 2018 and May 2019. The study cases were patients of both sexes, aged between 18 and 65 years, who were referred to the temporomandibular disorder outpatient clinic of our department. The inclusion criteria were the diagnosis of a localized MM of more than one year of evolution in the temporomandibular area and the presence of at least one active TrP located in the masticatory musculature. Individuals with one or more of the following conditions were excluded: inflammatory processes at the injection site; concomitant treatment with aminoglycosides or quinolones; pregnancy; lactation; dentofacial deformities; previous mandibular trauma; chronic neuromuscular diseases; an increased risk of bleeding; migraine or tension headaches; odontogenic infections; fibromyalgia syndrome; uncontrolled metabolic disorders; or significant depressive disorders. 

Examination of the masticatory muscles centered on the temporalis, masseter, and lateral pterygoid muscles bilaterally, combining pressing, sliding and pinching movements to detect the presence of TrPs and areas of myalgia, according to the technique already known and described previously [8,10]. Pre-puncture EMG was performed to determine the muscle masses to be treated and to detect the pattern of muscle involvement at rest and during voluntary movement. A ten-channel Medelec Synergy device with Ambu^®^ Neuroline Inoject 35 mm × 0.40 mm 27G Ambu^®^ Neuroline needle electrodes was used. The computer version used for the EMG studies in this study was 22.1.1.153.

Patients with chronic MM were randomly assigned to two groups. Group 1 received a BTA injection (BTA group) and group 2 received percutaneous electrolysis (PNE group) applied using a specific Epimedical^®^ device that produces a modulated galvanic current. Assessments were performed before treatment (day 0) and 28, 60, and 90 days after treatment. The patients were assigned to one of two treatment groups using a random number generator. Each procedure was performed in a single session in order to assess its therapeutic indication, efficacy, and the safety of the treatment. 

**Figure 2:**
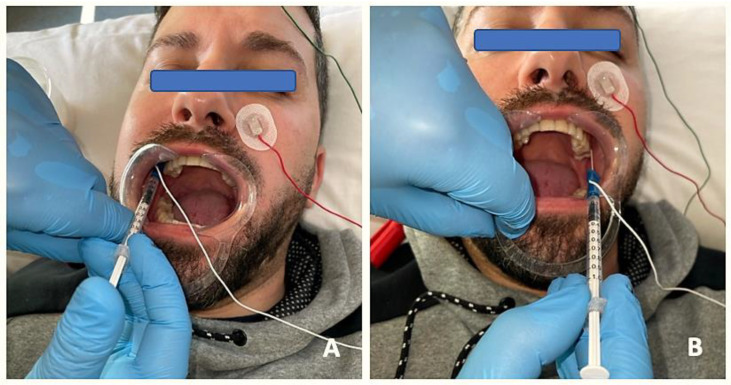
EMG prior to BTA infiltration to detect specific patterns of muscle involvement through insertion of needle electrodes into the lateral pterygoid muscle and injection of BTA on the right side (**A**) and on the left side (**B**).

**Figure 3:**
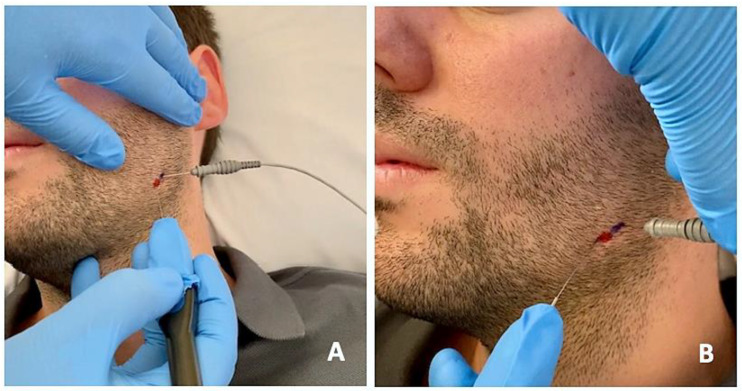
Percutaneous electrolysis with direct puncture of the affected masticatory muscles using a specific device (Epimedical^®^) that produces a modulated galvanic current (**A**). On the patient’s skin, the percutaneous needle puncture site is marked in red, and the placement of the EMG needle is marked in blue (**B**).

The protocol was the same in the two groups (Figure 2 and Figure 3): (1) manual localization by palpation of the muscle mass and, after performing EMG, intramuscular puncture. EMG equipment: ten-channel Medelec Synergy with Ambu^®^ Neuroline Inoject 27G needle electrodes (35 mm × 0.40 mm) and Ambu^®^ Neuroline ground surface electrodes. (2) Muscle puncture: prior to puncture and the injection of the different muscle masses, the area was disinfected with 90° alcohol. (2A) For the BTA group, 1 mL of BD U-100 insulin syringes with decimal scale graduation were used. (2B) For the PNE group, sterile Agupunt^®^ stainless steel needles (40 × 0.25 mm) were used. (3) Procedure. (3A) For the BTA group, 100 units of onabotulinumtoxin A (Botox^®^) were diluted in 2.50 mL of saline solution to obtain 4 units per 0.1 mL, which were injected into two points of the temporalis muscle (total amount of 0.2 × 2 = 0.4 mL or, more precisely, 16 units), two points in the masseter muscle (total amount of 0.2 × 2 = 0.4 mL or 16 units), and one point in the lateral pterygoid muscle (total amount of 0.4 × 1 = 0.4 mL or 16 units). Taking into account the dilution used, the dose administered was 96–100 units, distributed between the different muscle masses bilaterally. (3B) For the PNE group, percutaneous electrolysis with deep dry needling was applied directly to the affected masticatory muscles with low-intensity parameters of 0.5 mA for 3 s and 3 times in a single session. Following the puncture, hemostatic compression was performed for 1 min. No muscle stretching was performed after the puncture.

### 5.3. Measurements

The primary parameters for assessing treatment efficacy were as follows: (1) pain at rest and during mastication using a visual analog scale (VAS, 10 cm) and (2) the amplitude of mandibular movements associated with mouth opening, lateral movements, and protrusion, measured with a Therabite^®^ ruler. The signs assessed as indicators of efficacy were a significant reduction in masticatory pain at rest and during mastication, recovery of normal ranges of mandibular opening, lateral and protrusive movements, and improvement in TMJ function. TMJ involvement was evaluated using a questionnaire consisting of a 100-point scale (with 0 being the worst and 100 being the best), evaluating pain (40 points), function (45 points), and chewing (15 points). TMJ impairment was assessed using a questionnaire consisting of a 100-point scale (with 0 being the worst and 100 being the best), assessing pain (40 points), function (45 points), and chewing (15 points). Secondary efficacy outcomes were the global efficacy assessments estimated by the patient and the observer using a four-point scale ranging from 0 (worst) to 4 (best). The patient and observer assessed the tolerability to treatment using a four-point scale (very poor = 0 points, poor = 1 point, fair = 2 points, good = 3 points, and excellent = 4 points). The type and frequency of adverse events were recorded at each visit.

### 5.4. Statistical Analysis 

The data were analyzed with IBM SPSS Statistics 20. The sample size was estimated expecting to find a decrease in two or more pain points in VAS, after injection of BTA (BTA group) or PNE puncture (PNE group), which had to be present within a month after treatment. Considering a significance level of 5% and equivalence limit of 1.35, a one-sided double-equivalence Student’s *t*-test based on two independent series was performed, providing us with 25 cases needed per group analyzed. Estimating a possible drop-out rate of 2%, a recruitment of 26 individuals per therapeutic procedure group was considered (*n* = 52 patients total). The data were first analyzed with a general statistical test using absolute and relative frequencies for qualitative variables and mean values, standard deviation (SD) or 50th percentile (P50; median = Me), or P25–P75 (interquartile range = IQR) for quantitative variables. Normality tests were established in each group using the Kolmogorov–Smirnov and Shapiro–Wilk tests. The Friedman test and Wilcoxon signed-rank test were used for intragroup comparative analysis at each checkpoint and for the intragroup comparative analysis at each consecutive checkpoint. For the intergroup comparative analysis, the Mann–Whitney U test was used. Values of *p* < 0.05 were considered to indicate statistical significance.

## Figures and Tables

**Table 1:** Demographic characteristics and variable description at baseline.

	BTA Group	PNE Group	*p*-Value
Age (mean years, range years)	39.11 (22–54)	41.96 (25–62)	0.373
Gender (female/male) (*n*)	19/7	18/8	0.547
Pain (VAS) (M ± SD)	6.50 (±1.06)	6.42 (±1.02)	0.827
Maximum interincisal opening (mm) (M ± SD)	37.81 (±5.65)	40.15 (±6.34)	0.196
Lateral-right (mm) (M ± SD)	5.08 (±1.74)	6.08 (±1.97)	0.085
Lateral-left (mm) (M ± SD)	5.54 (±1.44)	6.15 (±1.51)	0.117
Protrusion (mm) (M ± SD)	5.19 (±1.62)	5.46 (±1.44)	0.497

Abbreviations: BTA = botulinum toxin group. PNE = percutaneous needle electrolysis. VAS = visual analog scale. M = mean. SD = standard deviation. Significance (*p*): Mann–Whitney test for intergroup comparative analysis. Results were considered significant (*p* < 0.05).

**Table 2:** Intergroup and intragroup analysis at each checkpoint.

Group	Day 0	Day 28	Day 60	Day 90	*p*-Value (2)
**Pain (VAS)**					
BTA (M ± SD)	6.50 (±1.06)	2.73 (±1.00)	2.42 (±1.23)	2.58 (±1.36)	<0.001 *
PNE (M ± SD)	6.42 (±1.02)	2.77 (±0.95)	2.58 (±0.85)	2.46 (±1.02)	<0.001 *
BTA vs. PNE **(*p*-value) (1)**	-	0.778	0.394	0.872	-
**Maximum interincisal opening (mm)**					
BTA (M ± SD)	37.81 (±5.65)	41.46 (±5.43)	41.88 (±5.16)	42.73 (±4.85)	<0.001 *
PNE (M ± SD)	40.15 (±6.34)	43.58 (±5.93)	44.15 (±5.60)	44. 69 (±5.30)	<0.001 *
BTA vs. PNE **(*p*-value) (1)**	-	0.171	0.067	0.104	-
**Lateral-right (mm)**					
BTA (M ± SD)	5.08 (±1.74)	6.88 (±1.77)	7.12 (±1.77)	7.19 (±1.60)	<0.001 *
PNE (M ± SD)	6.08 (±1.97)	7.00 (±1.69)	7.08 (±1.71)	7.12 (±1.79)	<0.001 *
BTA vs. PNE **(*p*-value) (1)**	-	0.757	0.970	1.000	-
**Lateral left (mm)**					
BTA (M ± SD)	5.54 (±1.44)	6.77 (±1.81)	7.04 (±1.92)	7.23 (±2.06)	<0.001 *
PNE (M ± SD)	6.15 (±1.51)	7.00 (±1.76)	7.12 (±1.81)	7.08 (±1.85)	<0.001 *
BTA vs. PNE **(*p*-value) (1)**	-	0.568	0.911	0.823	-
**Protrusion (mm)**					
BTA (M ± SD)	5.19 (±1.62)	6.23 (±1.75)	7.00 (±1.62)	7.04 (±1.70)	<0.001 *
PNE (M ± SD)	5.46 (±1.44)	5.84 (±1.26)	6.54 (±1.36)	6.54 (±1.24)	<0.001 *
BTA vs. PNE **(*p*-value) (1)**	-	0.600	0.287	0.349	-

Abbreviations: M = mean. SD = standard deviation. VAS = visual analog scale. BTA = botulinum toxin group. PNE = percutaneous needle electrolysis. (1) Significance (*p*): Mann–Whitney test for intergroup comparative analysis between groups. (2) Significance (*p*): Friedman test for intragroup comparative analysis. Results were considered significant (*p* < 0.05) *.

**Table 3:** Intragroup analysis every two consecutive checkpoints.

	Δ 0–28	Δ 28–60	Δ 60–90
Group	*p*-Value	*p*-Value	*p*-Value
**Pain (VAS)**			
BTA	<0.001 *	0.273	0.667
PNE	<0.001 *	0.390	0.707
**Maximum interincisal opening (mm)**			
BTA	0.002 *	0.452	0.107
PNE	0.001 *	0.390	0.283
**Lateral-right (mm)**			
BTA	0.006 *	0.420	0.629
PNE	0.018	0.555	0.914
**Lateral left (mm)**			
BTA	0.012	0.485	0.788
PNE	0.006 *	0.519	0.788
**Protrusion (mm)**			
BTA	0.076	0.013	0.957
PNE	0.004 *	0.420	1.000

Abbreviations: Δ = change. VAS = visual analog scale. BTA = botulinum toxin group. PNE = percutaneous needle electrolysis. Significance (*p*): Wilcoxon test for intragroup comparative analysis every two consecutive checkpoints. Results were considered significant (*p* < 0.05) *.

**Table 4:** Intragroup analysis at each checkpoint.

Group	Day 0	Day 28	Day 60	Day 90	*p*-Value
	M (±SD)	M (±SD)	M (±SD)	M (±SD)	
**100-point questionnaire (0–100)**					
BTA	70.83 (±14.62)	79.37 (±15.35)	80.25 (±14.52)	86.65 (±13.60)	<0.001 *
PNE	67.63 (±14.48)	85.85 (±14.23)	85.85 (±14.23)	85.73 (±14.21)	<0.001 *
**Patient efficacy outcomes (0–4)**					
BTA	-	3.23 (±0.71)	3.12 (±0.65)	3.08 (±0.68)	0.074
PNE	-	3.19 (±0.56)	3.23 (±0.58)	3.15 (±0.61)	0.607
**Observer efficacy outcomes (0–4)**					
BTA	-	3.46 (±0.50)	3.42 (±0.50)	3.46 (±0.50)	0.846
PNE	-	3.23 (±0.58)	3.19 (±0.56)	3.19 (±0.56)	0.717
**Patient tolerance outcomes (0–4)**					
BTA	-	3.38 (±0.69)	3.46 (±0.64)	3.42 (±0.70)	0.368
PNE	-	3.04 (±0.77)	3.04 (±0.77)	3.00 (±0.80)	0.717
**Observer tolerance outcomes (0–4)**					
BTA	-	3.27 (±0.72)	3.31 (±0.73)	3.35 (±0.74)	0.223
PNE	-	3.04 (±0.77)	3.08 (±0.79)	3.04 (±0.77)	0.368

Abbreviations: M = mean. SD = standard deviation. VAS = visual analog scale. PNE = percutaneous needle electrolysis. BTA = botulinum toxin group. Significance (*p*): Friedman test for intragroup comparative analysis. Results were considered significant (*p* < 0.05) *.

## Data Availability

The data presented in this study are available by contacting the corresponding author upon reasonable request.
